# Supplementary material for: T cells in the brain enhance neonatal mortality during peripheral LCMV infection
Source: PLoS Pathog. 2021 Jan 5;17(1):e1009066. doi: 10.1371/journal.ppat.1009066 (PMC7785120; doi:10.1371/journal.ppat.1009066)
Supplement: Attachment — Submitted filename: Neonate paper point by point response 10.12.20.docx [file ppat.1009066.s001.docx]

October 3rd, 2020

PPATHOGENS-D-20-00402REVISED
Kenney et al

We are grateful to the reviewers and the Editor for their constructive comments and have revised the manuscript to address them. Specific responses to each of the reviewers’ comments are provided below:

Part I - Summary

**Reviewer #1:** The outcome to systemic virus infection in adults can vary depending on inoculum dose, dissemination, and resulting T cell responses, which can either control infection or cause pathogenesis. Neonatal mice have immature innate and adaptive immune systems, so it is unknown how infection dose affects outcome to infection. Herein, 1-week old mice were found to be highly susceptible to very low doses (5 PFU) of LCMV-Armstrong, a viral strain that does not cause pathogenesis in adult mice. LCMV-Armstrong was not controlled early after challenge, allowing for dissemination into the brains of neonatal mice. While CD8+ T cells in the spleen failed to express antiviral cytokines, CD8+ T cells in the brain remained proliferative and were protected from exhaustion, leading to perforin dependent CD8+ T cell-mediated immunopathology in the brain. Neonates from LCMV-immune moms were passively protected and showed lower initial burdens of LCMV-Armstrong and generated strong peripheral CD8+T cell responses. The virus did not disseminate to the brain in the passively protected mice, allowing for survival. Infection of neonates with Pichinde virus that replicates to only low levels failed to cause pathogenesis in neonates. Overall, the data show that infection dose impacts outcome to infection in neonates; the weakened initial immune response allows for virus dissemination to the brain where CD8+ T cells cause pathogenesis; passive immunity that effectively lowers early virus growth allows neonates to generate protective CD8+ T cell responses. Overall, this is a well-written manuscript describing several very interesting observations though no new mechanistic insights are highlighted.

Reviewer #2: “T cells in the brain enhance neonatal mortality during peripheral LCMV infection” is a manuscript by Kenney and colleagues that seeks to understand the neonatal immune response to viral infection. The authors demonstrate that neonatal mice have limited numbers of CD4+ and CD8+ T cells that increase over time. They then show low dose Armstrong infection is lethal to 7 day old animals, but mice older than 12 days are protected. This effect is observed between 5-500 pfu of the Armstrong strain. Viral titers in these animals are higher than adult animals infected with higher doses. Genetic depletion of T cells rescues the lethality, while singular depletion of either CD4+ or CD8+ T cells does not. Antibody-mediated neutralization of IFNg and TNFa is not sufficient for rescue, but loss of perforin is. The authors then examine immune responses in the brains of surviving animals and find a delay the antigen-specific CD8+ T cell response in neonates. The virus-specific cells in the brains appear to have more PD-1. Two other neonatal models are used: a LCMV-immune maternal model and pichinde virus infection. While these studies are in an important area there are some major problems that preclude publication in the current form.

Reviewer #3: This manuscript examines the outcome of acute LCMV infection in neonatal mice infected at 1 week after birth. Using the acute and normally nonlethal Armstrong strain of LCMV, the authors show that ~80% of neonatal mice infected with as low as 5 PFU of virus succumb to infection between days 8-14 post-infection. Compared to adult mice, the neonatal mice failed to control initial virus replication in the Kidney, Lung and Liver. TCRb-deficient mice were completely protected from LCMV-induced mortality indicating T cells play an important role in mediating the lethal immunopathology. Antibody-mediated depletion of either CD4 or CD8 T cells resulted in some enhanced survival with the greater effect observed following CD8 T cell depletion. Additional studies using perforin-deficient mice demonstrated that complete survival of neonatal mice lacking perforin suggesting a role for perforin-mediated lysis by CD8 T cells in the immunopathology. A wealth of additional data is provided indicating that the CD8 T cells exhibit a partially exhausted phenotype in the neonatal mice and that virus is able to enter the brain in neonatal mice and that CD8 T cells can be isolated from the brains that are more functional than those found in the periphery. Neonatal mice born from LCMV-immune mothers were protected from lethal LCMV-induced disease via the maternal antibodies they received. In addition, neonatal mice infected with a less virulent arenavirus, Pichinde virus, did not exhibit lethal disease. Overall, this manuscript is well written and the authors provide convincing data in support of their primary conclusions.

**Author’s response**:

**We thank the reviewers for their positive comments about our manuscript.**

Part II – Major Issues:

Reviewer #1: Concerns/comments.

1. Figures 4 and 6 show that neonatal mice have CD8+ T cells in the periphery that are reduced in magnitude and can develop an exhaustion phenotype, whereas T cells in the brain are protected from T cell exhaustion following Armstrong. This seems to be a major finding but is not further explored or explained mechanistically. What is special about the neonatal brain that would allow T cells to escape exhaustion? Do neonates show T cell exhaustion in the brain following LCMV-Clone13 (Figure 6CD)?

**Author’s response**:

**At this time, we do not know if there is anything immunologically different about the brain environment that would allow the CD8 T cells to survive in the brain and not in the periphery. This is a highly complex issue and would be the topic of a completely new study. We do suggest in the manuscript that it may just be a matter of the kinetics of the virus which replicates at high levels in the kidney, liver and lung as early at day 2 of infection but does not reach significant levels in the brain until day 6 (because of the blood brain barrier). This may give enough time for the CD8 T cells that have come from the peripheral circulation to become fully functional before the viral load in the brain starts to increase at day 6 when the peripheral CD8 T cells are exhausted and losing control of the virus. This is discussed more fully in the discussion now on page 16 line 369-79.**

**“Interestingly, the brain may be a sight where T cells are protected from clonal exhaustion. At this time, we do not know if there is anything immunologically different about the brain environment that would allow the CD8 T cells to survive in the brain and not in the periphery. It is possible that the CD8 T cells may be protected from exhaustion due to the delayed kinetics of high viral loads in the brain compared to the periphery where CD8 T cells were partially exhaust. It may just be a matter of the kinetics of the virus which replicates at high levels in the kidney, liver and lung as early at day 2 of infection but does not reach significant levels in the brain until day 6 (because of the blood brain barrier). This may give enough time for the CD8 T cells that have come from the peripheral circulation to become fully functional before the viral load in the brain starts to increase at day 6 when the peripheral CD8 T cells are exhausted and losing control of the virus.”**

**We did not perform T cell studies in the brain of the high dose LCMV clone13 infected mice as all of the neonates survived. This suggested to us that as all of the T cells are gone from the peripheral circulation there would be none available to traffic to the brain. The high dose LCMV cl13 studies were only done as controls to demonstrate that the neonatal immune response was capable of full clonal exhaustion.**

Reviewer #2: 1. It is not novel, even within the limited field of LCMV immunobiology, that the system can have different effects (memory, exhaustion, death) with different strains, doses and backgrounds. The novel finding here is that 7 day old mice undergo immunopathology but the mechanisms are poorly explored. The authors state that cytolysis should be shut off, but no data are provided. They should either test in a Cr release assay, or as a surrogate perform CD107a and b staining and determine if this is altered.

2. Outside of PD-1, there really are no markers of exhaustion analyzed. What about other inhibitory receptors? Levels of Tox?

**Author’s response**:

**We agree that the important finding in these studies is that low dose LCMV infection is highly lethal in 7 day old neonatal mice. Our studies definitively show by using genetically modified mice such as TCRb KO mice and perforin KO mice that perforin-producing T cells are important in mediating the immunopathology. Antibody depletion studies suggested CD8 T cells play a role. Treatment with anti IFNg and Enbrel (anti-)TNF show that these cytokines do not appear to be an obligate requirement.**

**There are extremely limited numbers of cells that can be obtained from the neonatal mice spleen. When we designed our experiments, we had to limit the number of antibody stainings which could be done. Since our previous research had shown that using an TNF/IFNg ratio gave us a highly quantitative measure of partial exhaustion (ref 1,2 below) we focused our studies on these two cytokines which are highly useful for quantifying T cell exhaustion.**

**Also, due to the limited number of cells we had available for staining we focused our exhaustion marker staining on the most well characterized marker, PD1 in LCMV induced CD8 T cell exhaustion.**

**Reference :**

(1) **M. Cornberg, L. L. Kenney, A. T. Chen, S. N. Waggoner, S.-K. Kim, H. P. Dienes, R. M. Welsh, L. K. Selin, Clonal exhaustion as a mechanism to protect against severe immunopathology and death from an overwhelming CD8 T cell response, *Front Immunol* 4, 475 (2013)**
(2) **S. N. Waggoner, M. Cornberg, L. K. Selin, R. M. Welsh, Natural killer cells act as rheostats modulating antiviral T cells, *Nature* 481, 394–398 (2012).**

3. The switching between different virus doses, sometimes even within a figure is extremely confusing.

Is there a way to use one dose?

**Author’s response**:

**We apologize that the use of two different doses of LCMV Armstrong (5 and 50 pfu) in neonatal mice causes confusion. However, many of these experiments were carried out in parallel before we had the complete results of the dose response mortality curve (Fig 1F). Since all 3 doses were equivalent in the induction of mortality in neonatal mice, it did not justify using and killing more mice to repeat some of the experiments. Our IACUC requires that we do everything possible to decrease the use of animals, especially for experiments that are as complex as these are to design and carry out where the pups for any one experiment have to be born at the same time and remain with their mothers throughout the experiments.**

4. Are the cells in surviving neonates at ~60 days memory or exhausted?

**Author’s response**:

**As indicated in the manuscript the neonatal mice that survive, clear the virus essentially by day 14 (Fig 2) and their T cell populations continue to increase as the mouse matures and they develop normal memory CD8 T cell frequencies (Fig 1A-D), and in that process develop equivalent LCMV-specific CD8 T cell responses to adults (Fig 5). We have added a sentence to the text in the results section to make this more clear (pg 10. ln. 221-24)**

**“However, the neonatal mice that survived, cleared the virus essentially by day 14 (Fig 2) and their T cell populations continued to increase as the mouse matures and they developed normal functional memory CD8 T cell frequencies (Fig 1A-D), and in that process develop equivalent LCMV-specific CD8 T cell responses to adults (Fig 5). “**

5. To this reviewer at least, the comparison of much higher doses in older animals for both LCMV and PiV is meaningless. Isn’t the appropriate comparison older mice with the low doses? Even if the titers are below the detection limit is this more relevant?

**Author’s response**:

**Our goal was to compare and contrast the immune responses of neonatal mice to the classic well-characterized adult LCMV and PICV models of infection.**

6. Conditions for CD3 stimulation of ICS need to be described.

**Author’s response**:

**We have added to the methods section on ICS that we used “5 μg/ml anti-CD3 (145-2C11)”**

**“Single cell suspensions were stimulated with 1uM of peptide or 5 μg/ml anti-CD3 (145-2C11), and incubated with Golgi plug (BD Bioscience) and recombinant human IL-2 for 4.5 hours at 37°C.”**

7. How total LCMV-specific response calculated needs to be described.

**Author’s response**:

**We have added the following statement to the figure legend of Figure 5.**

**“The total LCMV frequency and number was calculated by summing the individual responses to LCMV epitopes NP396, GP33, GP276 and NP205 based on intracellular cytokine assay results.”**

**We have also added the following statement to the figure legend of Figure 9.**

**The total PICV frequency and number was calculated by summing the individual responses to PICV epitopes NP38, NP122 and NP205 based on ICS assay results.**

8. Are Figure 6A and B spleen or brain, it is very difficult to determine.

**Author’s response**:

**Figure Fig6A and Fig 6B are spleen. We have clarified this by adding to the figure legend:**

**“A-B. LCMV Armstrong-specific CD8 T cells from the spleens of neonatal mice (50 PFU) and adult mice (5x10^4^ PFU) were examined on day 9 post infection for PD-1 expression (A.) and TNF production (ratio of IFNγ+TNF+ cells to IFNγ-only+ cells) (B).”**

9. Figure 1G and 7B have confusing labeling. Why not add appropriate tissue to the right of the boxes?

**Author’s response**:

**This has been done.**

10. Do the mothers in the LCMV immune experiment have neutralizing antibodies? This data should be shown.

**Author’s response**:

**As female mice who have been infected with LCMV are known to develop antibodies (REF 33 in manuscript) to LCMV, we did not measure the antibody levels.**

11. All viral titer figures need detection limit added.

**Author’s response**:

**The detection limit of the plaque assay for kidney, lung and brain is 1 log_10_ ; the liver is 2 log_10_. This has been added to figure legends.**

12. There are a lot of typographical mistakes in the manuscript. In multiple places morality is used instead of mortality. An arrow guiding the reader to pertinent pathology would be helpful.

**Author’s response**:

**We have edited the manuscript correcting typos and grammatical structure. This is evident in the tracked version of the manuscript. Arrows hasve been added to the pathology figures.**

13. The figure legends 1 and others, refer to “the same day 7 50 pfu group is shown in A and B for comparison”.

**Author’s response**:

**Since this point was confusing and not essential to the manuscript we have removed this reference.**

14. References 21 and 22 do not demonstrate three aa change between Armstrong and Clone 13!

**Author’s response**:

**We apologize for the error and have now revised that section of the manuscript to read (pg 3-4; ln. 70-73):**

**“LCMV clone 13 is a clonal strain isolated from a mouse persistently infected with LCMV Armstrong (14) and has a single amino acid distinct from the Armstrong strain (21, 22). This mutation alters the affinity of the LCMV clone 13 for the α-distroglycan receptor allowing it to rapidly systemically infect mice (21, 22).”**

15. The order of exhaustion described the authors Il-2 then IFNg then TNFa is not correct in Clone 13. See reference 15 for correct order!

**Author’s response**:

**We apologize for this error and have now corrected this. The sentences now read (pg4 in. 78-80) .**

**“The loss of cytokine production occurs in the order of IL-2, then TNF and then IFNγ, and clonal exhaustion finally drives apoptosis of antigen-specific T cells (15). Cytotoxic function is also lost early approximately at the same time as IL-2 production is lost.”**

Reviewer #3: None

Part III – Minor Issues: Editorial and Data Presentation Modifications

Reviewer #1:

2. Figure 8J-K is not described.

**Author’s response**:

**We apologize for this omission. For clarity we have divided Figure 8 into two separate figures with Figure 8A-D still being focused on viral load, while previous figure 8 E-K are now figure 9 A-F focus on kinetics and size of PICV-specific CD8 T cell responses. We have added to the text the following sentence. (page 14, ln 319-20)**

**“There also was no evidence of CD8 T cell exhaustion based on TNF+INFg+:IFNg ratio or PD1 expression ( Figure 9E&F)(previously Figure 8J&K).”**

3. Discussion. Reference 33 showed that maternal antibody can protect neonates during infection and the protection depended on b2m expression by the neonates and the neonates need T cells. While it is correct that virus-specific CD8+ T cells were not quantified, that paper established a role for CD8+ T cells in order for neonates to be passively protected. The effect of maternal antibody on virus loads was measured in that paper. Please clarify.

**Author’s response**:

**We have clarified our discussion of this study in the text. This section now reads (pg 17, ln 383-88)**

**“Maternal antibody from LCMV Armstrong (strain 4) immunized mothers had a measurable effect on decreasing viral load and blocked the development of persistent LCMV infection in neonatal mice less than 24 hours old (33). In that study the maternal antibody protection depended on b2m expression by the neonates and the neonates need T cells. Although the virus-specific CD8 T cells were not quantified, that study did establish a role for CD8 T cells in order for neonates to be passively protected.”**

4. It would be helpful to indicate in each legend where LCMV-Armstrong was used, rather than just “LCMV”, and specify that T cells from the spleen were analyzed.

**Author’s response**:

**We have made the requested modifications to the text in the legends and hope this increases clarity.**

5. Figure 5A,5C legend. Which epitopes were assessed ?

**Author’s response**:

**We have added the following statement to the figure legend of Figure 5.**

**“The total LCMV frequency and number was calculated by summing the individual responses to LCMV epitopes NP396, GP33, GP276 and NP205 based on intracellular cytokine assay results.”**

6. In reference to Fig5A-C, the text says “….lower than adults, at 89.5% and 55%....”. It would be clearer to refer to the percentages in the plot. 03vs2 and 30 vs 12

**Author’s response**:

**We have changed this sentence to now read (pg 9, ln 212-13).**

**“The frequency of the total CD8 T response was also lower in neonates than in adults, at 2% vs 32% and 12% vs 30%, at days 9 and 14, respectively.”**

Reviewer #2: (No Response)

Reviewer #3: A few minor points:

1. Third sentence of the last paragraph of the introduction, I believe the authors meat "Mortality" and not "Morality."

**Author’s response**:

**We have edited the manuscript correcting typos and grammatical structure. This is evident in the tracked version of the manuscript.**

2. Were combined CD4 and CD8 T cell depletions performed? If there were done, this would be a nice addition to Fig3b.

**Author’s response:**

**We did not do a combined CD4 and CD8 depletion.**

3. Were statistics performed on the survival curves shown in Fig 3A and 3E? If so, these should be added to the figure.

**Author’s response:**

**These were highly significant and p<0.001 (***) values were added.**

4. Was the function of CD4 T cells assessed? If so, this would be a nice addition to the manuscript.

**Author’s response:**

**Unfortunately, due to very limited numbers of cells that can be obtained from neonatal spleens we were only able to do CD8 T cell studies.**
